# An observational study reveals that neonatal vitamin D is primarily determined by maternal contributions: implications of a new assay on the roles of vitamin D forms

**DOI:** 10.1186/1475-2891-12-77

**Published:** 2013-06-07

**Authors:** Spyridon N Karras, Iltaf Shah, Andrea Petroczi, Dimitrios G Goulis, Helen Bili, Fotini Papadopoulou, Vikentia Harizopoulou, Basil C Tarlatzis, Declan P Naughton

**Affiliations:** 1 Unit of Reproductive Endocrinology, First Department of Obstetrics and Gynecology, Medical School, Aristotle University of Thessaloniki, Thessaloniki, Greece; 2 School of Life Sciences, Kingston University London, London, UK; 3 Department of Endocrinology, Diabetes and Metabolism, Panagia General Hospital, Thessaloniki, Greece

**Keywords:** Pregnancy, LC-MS/MS, Vitamin D epimer, Assay, 25(OH)D

## Abstract

**Background:**

Vitamin D concentrations during pregnancy are measured to diagnose states of insufficiency or deficiency. The aim of this study is to apply accurate assays of vitamin D forms [single- hydroxylated [25(OH)D_2_, 25(OH)D_3_], double-hydroxylated [1α,25(OH)_2_D_2_, 1α,25(OH)_2_D_3_], epimers [3-epi-25(OH)D_2_, 3-epi-25(OH)D_3_] in mothers (serum) and neonates (umbilical cord) to i) explore maternal and neonatal vitamin D biodynamics and ii) to identify maternal predictors of neonatal vitamin D concentrations.

**Methods:**

All vitamin D forms were quantified in 60 mother- neonate paired samples by a novel liquid chromatography -mass spectrometry (LC-MS/MS) assay. Maternal characteristics [age, ultraviolet B exposure, dietary vitamin D intake, calcium, phosphorus and parathyroid hormone] were recorded. Hierarchical linear regression was used to predict neonatal 25(OH)D concentrations.

**Results:**

Mothers had similar concentrations of 25(OH)D_2_ and 25(OH)D_3_ forms compared to neonates (17.9 ± 13.2 vs. 15.9 ± 13.6 ng/mL, p = 0.289) with a ratio of 1:3. The epimer concentrations, which contribute approximately 25% to the total vitamin D levels, were similar in mothers and neonates (4.8 ± 7.8 vs. 4.5 ± 4.7 ng/mL, p = 0.556). No correlation was observed in mothers between the levels of the circulating form (25OHD_3_) and its active form. Neonatal 25(OH)D_2_ was best predicted by maternal characteristics, whereas 25(OH)D_3_ was strongly associated to maternal vitamin D forms (R^2^ = 0.253 vs. 0.076 and R^2^ = 0.109 vs. 0.478, respectively). Maternal characteristics explained 12.2% of the neonatal 25(OH)D, maternal 25(OH)D concentrations explained 32.1%, while epimers contributed an additional 11.9%.

**Conclusions:**

By applying a novel highly specific vitamin D assay, the present study is the first to quantify 3-epi-25(OH)D concentrations in mother - newborn pairs. This accurate assay highlights a considerable proportion of vitamin D exists as epimers and a lack of correlation between the circulating and active forms. These results highlight the need for accurate measurements to appraise vitamin D status. Maternal characteristics and circulating forms of vitamin D, along with their epimers explain 56% of neonate vitamin D concentrations. The roles of active and epimer forms in the maternal - neonatal vitamin D relationship warrant further investigation.

## Introduction

Vitamin D insufficiency and deficiency has been associated with a wide spectrum of diseases, ranging from neurological disorders to chronic inflammatory conditions [1]. The resurgence of rickets in some Western countries highlights the potential risks of not gaining sufficient vitamin D through diet, supplementation or exposure to sunlight [2,3]. Vitamin D deficiency is frequently defined as serum concentrations less than 20 ng/mL with concentrations between 21–29 ng/mL treated as insufficiency and greater than 30 ng/mL as sufficient [4-7]. Recent studies attest to widespread insufficiency of vitamin D in many Western nations, namely the UK, USA and other European countries, including Greece [5,6,8]. Vitamin D deficiency during pregnancy has been associated with maternal morbidity, including gestational diabetes [9] and an increased rate of caesarean section [10]. Likewise, for the neonate, there is a putative association with being small-for-gestational age (SGA) [11]. Finally, as far as children are concerned, impaired neurocognitive development [12] and skeletal problems, such as reduced bone mineral content [13] have been reported.

A recent report details the importance of maternal circulating vitamin D concentrations in determining neonatal circulating vitamin D [14]. The authors compared the contributions of genetic factors to maternal vitamin D levels and found that 19% of neonatal circulating vitamin D levels are predicted by the latter with genetics having little influence. The recent report of a lack of significant relationship between circulating 25(OH)D and the highly active 1α,25-(OH)_2_D concentrations in a meta-analysis of mother-neonate studies suggest that measurement of vitamin D concentrations should go beyond the routinely measured 25(OH)D forms [15]. Many studies have relied on questionable assays to assess concentrations of the various forms of vitamin D [16,17]. Given the complexities involved in rigorous assessment of vitamin D analogues, a novel assay was recently introduced to differentiate and quantify the circulating precursors and active forms from biologically inactive epimers [18,19]. It is envisaged that the role of vitamin D in disease prevention and treatment can be further elucidated with the accurate measurement of all forms of vitamin D, including epimers.

The primary aim of this study was to determine serum (mothers) and umbilical cord (neonates) concentrations of all vitamin D forms [single-hydroxylated [25(OH)D_2_, 25(OH)D_3_], double-hydroxylated [1α,25(OH)_2_D_2_, 1α,25(OH)_2_D_3_], epimers [3-epi-25(OH)D_2_, 3-epi-25(OH)D_3_]], in a Northern Greece cohort of pregnant women at term and their neonates, by applying a novel highly specific and accurate assay. A secondary aim was to predict neonatal vitamin D concentrations by means of maternal parameters.

### Subjects and methods

#### Subjects

The study was conducted from January 2011 until December 2011. Pregnant women were recruited from the Maternity Unit of the First Department of Obstetrics and Gynaecology, Aristotle University, Thessaloniki, Greece. Inclusion criterion was full-term pregnancy (37th -42th gestational week). Maternal exclusion criteria were primary hyperparathyroidism, secondary osteoporosis, liver disease, hyperthyroidism, nephrotic syndrome, inflammatory bowel disease, rheumatoid arthritis, osteomalacia, morbid obesity, diabetes in pregnancy, age < 18 year and use of medications affecting calcium (Ca) or vitamin D status. Neonatal exclusion criteria were being small-for-gestational age (SGA) and presence of severe congenital anomaly. Informed consent was obtained from all mothers. The protocol received approval from the Bioethics Committee of Aristotle University of Thessaloniki, Greece.

#### Demographics and diet

At enrolment, demographic and social characteristics were recorded. Ca and vitamin D dietary intake during the last month of pregnancy were assessed through a validated, semi- quantitative, food frequency questionnaire that includes 150 foods and beverages [20]. For each dietary item, participants were asked to report their frequency of consumption and portion size. From these data, calculations were made for estimations of consumed quantities (in g per day) and total energy intake (in kcal per day), on the basis of a food composition database, modified to accommodate the particularities of the Greek diet [21].

#### Biochemical and hormonal assays

Blood samples were obtained from mothers by antecubital venipuncture 30–60 minutes before delivery. Umbilical cord blood was collected immediately after clamping, from the umbilical vein. Serum and umbilical cord specimens were stored at -20°C prior to analysis for the following parameters: Ca, phosphorus (P), parathyroid hormone (PTH), vitamin D_2_, vitamin D_3_, 25(OH)D_2_, 25(OH)D_3_, 1α,25(OH)_2_D_2_, 1α,25(OH)_2_D_3_, 3-epi-25(OH)D_2_ and 3-epi-25(OH)D_3_. Serum Ca and P determinations were performed using the Cobas INTEGRA clinical chemistry system (D-68298; Roche Diagnostics, Mannheim, Germany). The inter- and intra-assay coefficients of variation (CVs) were 0.99% and 3.5% for Ca, and 1.3% and 2.5% for P, respectively. PTH determinations were performed using the electrochemiluminescence immunoassay ECLIA (Roche Diagnostics GmbA, Mannheim, Germany). Reference range for PTH was 15–65 pg/mL, functional sensitivity 6.0 pg/mL, within-run precision 0.6 - 2.8% and total precision 1.6 - 3.4%. Using the novel assay, a total of eight forms of vitamin D were quantified by liquid chromatography tandem mass spectrometry (LC-MS/MS) with lower limits of quantification (LLOQ) as follows: vitamin D_2_ (0.5 ng/mL), vitamin D_3_ (0.5 ng/mL), 25(OH)D_2_ (0.5 ng/mL), 25(OH)D_3_ (0.5 ng/mL), 1α,25(OH)_2_D_2_ (0.015 ng/mL), 1α,25(OH)_2_D_3_ (0.015 ng/mL), 3-epi-25(OH)D_2_ (0.01 ng/mL) and 3-epi-25(OH)D_3_ (0.015 ng/mL). Briefly, the assay involves a chiral column in tandem with a rapid resolution microbore column along with liquid-liquid extraction. The method is fully validated using quality controls at four different concentration levels (QCL, QCM, QCH, LLOQ). Quality controls were calculated after chromatographically separating the epimers, isobars and other analogues. The same concentrations were recovered from spiked quality controls prepared in house. The accuracy of the assay was also double checked using DEQAS and Chromsystem quality controls. Full method validation parameters have been reported previously [18,19]. Maternal vitamin D deficiency was defined as serum concentrations ≤ 20 ng/mL, insufficiency as 21–29 ng/mL and sufficiency as ≥ 30 ng/mL.

#### UVB measurements

Ultraviolet B (UVB) radiation includes wavelengths from 280 to 320 nm. UVB data for the broad geographical region of Thessaloniki, Greece were collected from the Section of Applied and Environmental Physics, Aristotle University of Thessaloniki. Daily integral of effective UVB radiation from sunrise to sunset (from 09:00 to 16:00) was used as the most representative parameter for UVB exposure. These hours were selected as they represent the beginning and the end of the working time for the majority of the population. Individual sunlight exposure was recorded for each participant during that period. Finally, mean UVB exposure during the previous 45 days (daily integral) before blood sample collection (estimated mean half-life of vitamin D) was calculated for each participant.

#### Statistical analysis

The dependent variables (DV) were the concentrations of circulating vitamin D_2_ and D_3_ in neonates. Adjusted body mass index (BMI) was calculated by adjusting the pre-delivery weight with the average expected weight gain based on the mother’s pre-pregnancy BMI. In cases below the limit of quantification (BLQ), a conservative zero value was imputed. Owing to large within group variances, vitamin D concentrations between mothers and neonates were compared using Wilcoxon Signed Rank test. ANOVA was used to compare the circulating vitamin D concentrations in neonates of mothers with deficient, insufficient and sufficient vitamin D status. To determine the explained variances by the independent variables (IV) in predicting the DV (neonatal serum vitamin D_2_ and D_3_, separately), two hierarchical linear regression analyses were used. In both models, in order to control for random differences between mothers (e.g. maternal age, number of previous live birth, UVB exposure and vitamin D), these variables were entered in the first block, followed by serum concentrations of 25OHD_2_ and D_3_, along with their corresponding epimers, individually. Meeting assumptions for the regression models were defined as follows: Durbin-Watson statistics (d) between 1.5 and 2.5 for auto-correlation of residuals and Variance Inflating Factor (VIF) < 5 for multi-colinearity, along with satisfactory normal P-P plot of regressions standardized residual. The level of significance was set as p < 0.05. All statistical analyses were conducted in SPSS v19 (SPSS Inc, Chicago, Ill).

## Results

The sample consisted of 60 pairs of Caucasian mothers and their neonates. Mean maternal age was 32.8 ± 5.2 years, 40% with previous live birth (31.7% primiparous and 8.3% multiparous). The mean pre-conception BMI was 22.2 ± 3.3 kg/m^2^ (range 16.1 - 31.6), adjusted BMI was 22.4 ± 4.3 kg/m^2^ (range 13.5 - 35.5). Thirty-six women were on Ca supplementation (range 250 – 1000 mg per day, with 32 on 500 mg per day) and none were on vitamin D supplementation. Of the 60 neonates, 67% were female. PTH, Ca, P concentrations of mothers and neonates, along with the estimated daily average intake of Ca and vitamin D, and UVB exposure are presented in Table 1.

### Maternal and neonatal vitamin 25(OH)D concentrations

**Figure 1:**
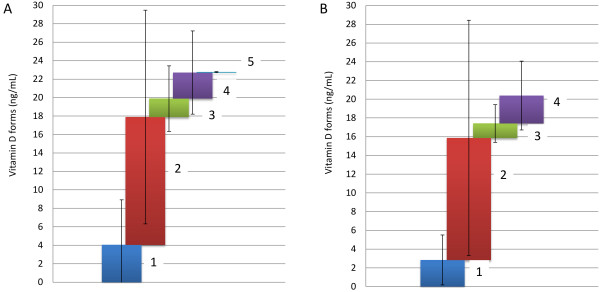
**Mean concentration of (A) maternal and (B) neonatal vitamin D forms.** 1: 25(OH)D_2_, 2: 25(OH)D_3_, 3: 3-epi-25(OH)D_2_, 4: 3-epi-25(OH)D_3_, 5: 1α,25(OH)_2_D_3_. Bars represent within-group standard deviations. Mean values for each analyte are presented in Additional file 1.

**Figure 2:**
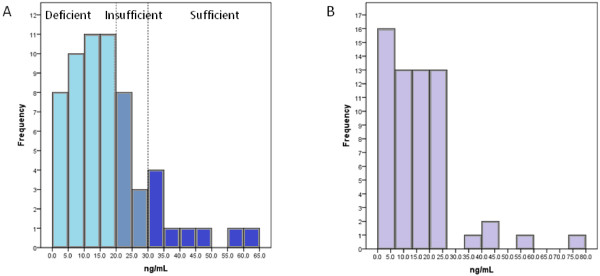
Frequency distribution of (A) maternal and (B) neonatal 25(OH)D concentrations.

Mothers had slightly, but not statistically significantly, higher concentrations of circulating vitamin D [25(OH)D_2_ and 25(OH)D_3_] compared to neonates (17.9 ± 13.2 vs. 15.9 ± 13.6 ng/mL, W = 771.0, p = 0.289) (Figure 1). The proportions of the mothers with sufficient, insufficient and deficient 25(OH)D concentrations are shown in Figure 2A. The frequency distribution revealed 40 women below 20 ng/mL, with a further 11 between 21–29 ng/mL, leaving a minority in the sufficient range. Notably, whilst the pattern of neonatal 25(OH)D concentration roughly followed the same of the mothers in the deficient and insufficient mother groups, it varied widely resembling uniform distribution in the group of mothers with sufficient vitamin D status. Although thresholds for neonatal serum vitamin D sufficiency are yet to be established, the frequency distribution of neonatal 25(OH)D concentrations (Figure 2B), followed the pattern of the maternal circulating levels, with the majority of the values being concentrated at the low end of the spectrum. The mean neonatal 25(OH)D concentrations in the three maternal groups were significantly different [12.5 ± 8.7 vs. 19.2 ± 9.1 vs. 26.6 ± 26.3 ng/mL,

**Table 1:** Measures of PTH, Ca, P concentrations of mothers and neonates, and daily average intake of Ca, vitamin D

	**Mother**		**Neonates**	
	**Range**	**Mean ± SD**	**Range**	**Mean ± SD**
Vitamin D ^a^ intake (μg/day)	0.35-5781.00	421.97 ± 1206.130	-	-
Ca intake ^a^ (mg/day)	111.0-1935.40	786.10 ± 360.240	-	-
UVB (wh/m^2^)	0.01-0.36	0.20 ± 0.11	-	-
PTH (mg/dL)^b^	19.00-85.40	36.91 ± 15.15	1.20-17.90	6.99 ± 2.78
Ca (mg/dL)^c^	4.20-9.60	8.56 ± 0.75	8.90-11.80	10.32 ± 0.62
P (pg/mL)^c^	1.40-5.00	3.58 ± 0.63	4.30-7.10	5.73 ± 0.58

^a^ mother n = 58.

^b^ mother n = 59, neonate n = 57.

^c^ mother n = 60, neonate n = 57.

 F(2,59) = 4.914, p =0.011] for neonates of mothers in the deficient, insufficient and sufficient group, respectively. This overall result was due to a difference between the deficient and sufficient groups (p =0.012), but not due to other comparisons (deficient vs. insufficient, p = 0.279 and insufficient vs. sufficient, p = 0.413).

### Proportions of vitamin D forms

**Figure 3:**
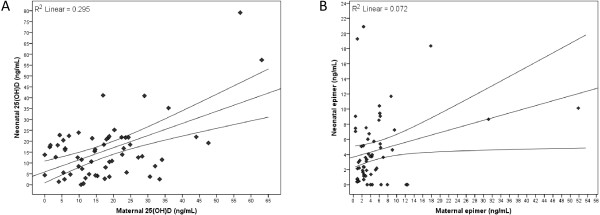
**Relationship between maternal and neonatal concentrations of (A) 25(OH)D [total 25(OH)D**_**2 **_**and 25(OH)D**_**3**_**] and (B) 3-epimers [total 3-epi-25(OH)D**_**2 **_**and 3-epi-25(OH)D**_**3**_**].** The fitted regression lines are accompanied by 95% confidence intervals.

Mean concentrations of vitamin D forms in mothers and neonates are illustrated in Figure 1. As far as the 1α,25(OH)_2_D_2_ and 1α,25(OH)_2_D_3_ forms are concerned, only 1α,25(OH)_2_D_3_ was measured in mothers, at a very low concentration (0.06 ± 0.06 ng/mL). The 1α,25(OH)_2_D_2_ form was below the limit of quantitation. In line with previous reports [15], no significant relationship was observed between maternal circulating forms [25(OH)D_3_] and the highly active 1α,25(OH)_2_D_3_ concentrations (r = -0.011, p = 0.931).The epimer concentrations (Figure 1) were similar in mothers and neonates (4.8 ± 7.8 vs. 4.5 ± 4.7 ng/mL, W = 1015.0, p = 0.462). Notably, 24.7% and 22.2% of the measured vitamin D forms were for inactive epimer forms for mothers and neonates, respectively. The 25(OH)D_3_ concentrations were higher compared to 25(OH)D_2_ levels (75.6 ± 22.2% in mothers and 75.9 ± 23.9% in neonates); thus, the ratios of 25(OH)D_3_: 25(OH)D_2_ were 3:1, approximately, for both mother and neonates. A summary table of means, standard deviations and mean measurement errors for the primary forms of vitamins D_2_ and D_3_ along with the active forms and their epimers is provided in Additional file 1. A positive correlation (r = 0.543, p < 0.001) was detected between maternal and neonatal 25(OH)D concentrations, whereas inactive [3-epi-25(OH)D] concentrations showed a weaker correlation (r = 0.268, p = 0.038) (Figure 3). The 25(OH)D and inactive [3-epi-25(OH)D] concentrations were positively correlated in the mothers (r = 0.528, p < 0.001) but not in the neonates (r = 0.142, p = 0.414). There was no significant correlation between 1α,25(OH)_2_D_3_ and 3-epi-25(OH)D concentrations.

### Maternal predictors of neonatal vitamin D concentrations

The hierarchical linear regression models for predicting neonatal 25(OH)D concentrations are detailed in Table 2. The correlation matrix (Pearson’s r) is provided in Additional file 2. In the majority of analyses, assumptions were met as defined. In the independent models (Table 2),

 the neonatal 25(OH)D_2_ concentrations were best predicted from maternal characteristics (R^2^ = 0.253), whereas 25(OH)D_3_ was strongly linked to maternal vitamin D forms (R^2^ = 0.478). Maternal serum concentrations of PTH, Ca and P together only explained a small proportion of the neonatal 25(OH)D_2_ (R^2^ = 0.046) and an even smaller part of the 25(OH)D_3_ (R^2^ = 0.013). Neonatal vitamin D concentrations were calculated as the sum of 25(OH)D_2_ and 25(OH)D_3_. Circulating neonatal vitamin D concentrations in newborns followed the pattern of predicting 25(OH)D_3_, with maternal 25(OH)D_2_ and 25(OH)D_3_ explaining 32.1% of the neonatal vitamin D variance and epimer forms contributing an additional 11.9%. Therefore, all four maternal vitamin D forms combined [25(OH)D_2_, 25(OH)D_3_, 3-epi-25(OH)D_2_, 3-epi-25(OH)D_3_] explained 44% of the neonatal vitamin D concentrations when controlled for other maternal characteristics such as age, UVB exposure, vitamin D and Ca intake and Ca, P and PTH concentrations. On the contrary, 1α,25(OH)_2_D_3_ did not make a contribution to the neonatal vitamin D concentrations. Predicting 25(OH)D in neonates, mother’s age showed statistical significance for the coefficients (β = -0.343). For 25(OH)D_2_, mother’s 25(OH)D_2_ concentrations showed statistical significance for the coefficients (β = 0.218) and 3-epi-25(OH)D_3_ (β = 0.596). These standardized β values can be used for weighting each individual’s measures on the IVs to obtain individual predicted score on the DV, respectively. Mother’s age was independent of vitamin D intake (r = -0.093, p = 0.483), negatively correlated with UVB exposure (r = -0.304, p = 0.019) and weakly negatively correlated with Ca intake (r = -0.244, p = 0.062).

## Discussion

### Maternal and neonatal vitamin 25(OH)D concentrations

The potential impact of vitamin D deficiency during pregnancy on maternal and neonatal health has attracted much interest in recent years. It has been suggested that maintaining adequate maternal stores of vitamin D during pregnancy is of vital importance for both mothers and neonates to ensure skeletal and extra-skeletal health.

The results of this study come mainly from a population of pregnant women with vitamin D deficiency or insufficiency. Although the study was not designed for this

**Table 2:** Hierarchical linear regression model for predicting neonatal 25(OH)D concentrations

		**DV: Neonatal 25(OHD)**_**2**_			**DV: Neonatal 25(OH)D**_**3**_		
**Step**	**IV (Maternal)**	**R**^**2**^	Δ**R**^**2**^	**SD β**	**R**^**2**^	Δ**R**^**2**^	**SD β**
1	UVB exposure			-0.243			-0.121
	Age			-0.343 *			-0.227 *
	Adjusted BMI			-0.172			0.032
	Vitamin D intake			0.082			0.143
	Ca intake	0.207	0.207 *	0.222	0.096	0.096	-0.018
2	Serum PTH			0.043			0.046
	Serum Ca			-0.267			0.169
	Serum P	0.253	0.046	-0.011	0.109	0.013	-0.161
3	25(OH)D_2_	0.254	0.002	0.040	0.267	0.158 **	0.271*
4	25(OH)D_3_	0.302	0.048	0.272	0.438	0.171 ***	0.218
5	Epi-25(OH)D_2_	0.303	0.000	0.124	0.518	0.080 **	-0.091
6	Epi-25(OH)D_3_	0.309	0.006	-0.179	0.586	0.068**	0.596 **
7	1α,25(OH)_2_D_3_	0.329	0.020	-0.147	0.587	0.001	0.032

* p < 0.05; ** p < 0.01, *** p < 0.001. BMI: body mass index, Ca: calcium, DV: dependent variable, IV: independent variable, P: phosphorus, PTH: parathyroid hormone, SD: standard deviation, UVB: ultraviolet B.

 purpose, a high prevalence of maternal vitamin D insufficiency and deficiency was detected, in a sunny European area, such as Northern Greece. A similar pattern of distribution between maternal and neonatal 25(OH)D concentrations was observed, with 25(OH)D_3_ being the most abundant circulating vitamin D form in both mothers and neonates. These results reflect previous reports of widespread vitamin D deficiency and insufficiency in Europe and the USA. However, the known cross-reactivity of many assays with the epimer forms suggests that levels reported in previous studies are overestimations. Furthermore, the conundrum of a mismatch between levels of the usually quantified circulating forms (25OHD) and the active form (1α,25-(OH)_2_D) [15] have been confirmed in this study, as no relationship was observed between 25(OH)D_3_ and 1α,25(OH)_2_D_3_ once epimers have been differentiated.

At this time, there is no documented benefit in measuring 25(OH)D_2_ and 25(OH)D_3_, separately; serum total 25(OH)D has been designated as the functional indicator of vitamin D status [22]*.* However, the ability to accurately measure serum concentrations of 25(OH)D_2_ and 25(OH)D_3_ brings new potential to both observational and intervention studies. On a physiological basis, it could be hypothesised that maternal vitamin D active forms have an impact on the newborn, which, to a great extent, depends on the mother to form its dynamic vitamin D equilibrium. Therefore, these findings confirm current concerns regarding the maintenance of adequate maternal vitamin D status during pregnancy, since the reflection of maternal concentrations of these forms explains 32.1% in neonates. It should be noted that data on 25(OH)D_2_ and 25(OH)D_3_ concentrations exclude epimer forms; thus, caution should be paid when comparing them to other studies [8].

Given that vitamin D_2_ is the only high-dose preparation available in many countries, potential differences in the ability of assays to accurately detect 25(OH)D_2_ and 25(OH)D_3_ are of clinical importance, in cases where supplementation is suggested. Moreover, when 25(OH)D results are reported as 25(OH)D_2_ and 25(OH)D_3_, vitamin D_2_ administration does reduce serum 25(OH)D_3_ concentrations [23]. Until the physiologic impact of this reduction, if any, is clarified by future studies, a low 25(OH)D_3_ value in the setting of ergocalciferol supplementation does not constitute vitamin D deficiency. LC-MS/MS and the potential of accurate measurement of both bioactive forms of vitamin D could offer a valuable tool in daily practice, in order to avoid data misinterpretation, especially in conditions like pregnancy.

### Proportions of vitamin D forms

The findings of this study, using a novel assay with the ability not just to exclude but also to measure vitamin D epimers demonstrated that epimers comprise approximately 25% of the measured vitamin D concentrations in both mothers and neonates, following similar patterns of distribution. The presence of both 3-epi-25(OH)D_2_ and 3-epi-25(OH)D_3_ forms have been previously reported in infants [24]. Our group has demonstrated the presence of 3-epi-25(OH)D_3_ form, in a small cohort of healthy adults [18]. These results were further confirmed in a larger study, in adults [25]. The present study is the first to quantify concentrations of the 3-epi-25(OH)D_2_ in both mothers and neonates. Large inter-individual variances in the epimer content were noted in vitamin D concentrations, ranging between 0% and 100% with 63.8% of the mothers and 67.8% of the neonates showing epimer to total circulating concentration of 25% or less. Thus, the epimer-adjusted concentration is only applicable to conclusions at the aggregated level (i.e. mothers, neonates) and should not be used for making judgments at the individual level, unless 3-epi-25(OH)D_2_ and 3-epi-25(OH)D_3_ are clearly separated and excluded from the 25(OH)D measurements. On the other hand, based on present results, it becomes evident that 1α,25(OH)_2_D_2_ and 1α,25(OH)_2_D_3_ have minor contributions to the sum of vitamin D measurements in both mothers and infants.

Based on these findings, it could be hypothesized that assays that do not separate the 3-epi forms or have significant cross-reactivity with the epimer will, most likely, report erroneously high concentrations for both infants and adults, as 3-epi constitutes a substantial fraction of total 25(OH)D. This assay limitation should be considered by clinicians measuring vitamin D status in infants and mothers. LC-MS/MS, by measuring vitamin D isoforms separately, provides a ‘clear-cut’ view of vitamin D status. By excluding epimer concentrations, it appears that the mean vitamin D concentration in term pregnancies is considerably below the sufficiency threshold. Although the sample size of the present study was small for drawing conclusions, the accurate measurement of active vitamin D metabolites could offer a valuable tool in the establishment of a novel, realistic view of vitamin D status during pregnancy.

### Maternal predictors of neonatal vitamin D concentrations

Based on our primary results, regarding the accurate proportions of vitamin D metabolites in maternal circulation, we further investigated if there is an ability to predict neonatal 25(OH)D concentrations from maternal parameters. Our analysis showed that, apart from being a reliable marker of vitamin D maternal status, 25(OH)D comprises a significant parameter in predicting neonatal 25(OH)D_3_ concentrations, which constitutes the major neonatal vitamin D form. The addition of certain maternal parameters could offer additional prognostic value in this process, specifically in neonatal 25(OH)D_2_ concentrations. The additional analytical capacity enhances the predictive power with the epimers contributing 11.9% to an overall 44% explained variances in active vitamin D concentrations in neonates. This result significantly exceeded previous reports of 19% in a twin study, which investigated genetic versus maternal vitamin D concentrations in determining offspring vitamin D concentrations [14]. Overall, the above findings regarding maternal vitamin D concentrations and other parameters could be useful in daily clinical practice, as a part of a predictive model for neonatal vitamin D concentrations, based on maternal parameters, which could contribute to the appropriate management of the major health issue of maternal vitamin deficiency during pregnancy.

### Advantages and disadvantages

The present study has three major advantages. First, the data incorporate specific and accurate measurement of seven out of eight forms of vitamin D, including vitamin D_2_ and D_3_, hydroxylated derivatives and epimer forms. To the best of our knowledge, these data are unique to the literature on pregnancy. Second, as routine assays do not allow differentiation among the full range of different vitamin D forms, this novel assay allowed for a very detailed approach to the complex vitamin D metabolism in the mother - newborn bipole. Third, the study allowed the control for maternal characteristics such as age, UVB exposure, dietary intake and PTH, Ca and P concentrations, which afforded separating the explained variances for the active vitamin D concentrations over and above the shared variances by maternal characteristics. In combination, we were able to explain 56.1% in the variances in neonatal 25(OH)D concentrations. Limitations of the study were its rather small sample size and its cross-sectional design, which prevented prospective data to be collected throughout pregnancy. Moreover, measurement of vitamin D-binding protein (VDBP), a significant parameter of vitamin D dynamics in pregnancy, was not feasible.

## Conclusions

This study provided evidence for i) maternal and neonatal vitamin 25(OH)D concentrations in a sunny European area, which proved to be sub-optimal, ii) 3-epi-25(OH)D_3_ and 3-epi-25(OH)D_2_ forms in both mothers and neonates, which contribute approximately 25% to the total vitamin D concentrations and iii) a relationship between maternal and neonatal concentrations leading to a prediction model. The accurate assay highlights a considerable proportion of vitamin D exists as epimers and there is a lack of correlation between the circulating and active forms. These results underscore the need for accurate measurements to appraise vitamin D status. The results, based on specific and accurate measurement, revealed that maternal characteristics and active forms of vitamin D, along with their epimers explain 56% of neonatal vitamin D concentrations. Further investigation, based on accurate measurements of vitamin D metabolites, is warranted to establish optimal concentrations during pregnancy, in an attempt to prevent maternal morbidity and developmental deficiencies.

## Abbreviations

1α,25(OH)2D2: 1-alpha-25-dihydroxyvitamin D_2_; 1α,25(OH)2D3: 1-alpha-25-dihydroxyvitamin D_3_; 25(OH)D2: 25-hydroxyvitamin D_2_; 25(OH)D3: 25-hydroxyvitamin D_3_; 3-epi-25(OH)D2: 3-epimer-25-hydroxyvitamin D_2_; 3-epi-25(OH)D3: 3-epimer-25-hydroxyvitamin D_3_; BLQ: Below the limit of quantification; Ca: Calcium; DV: Dependent variables; BMI: Body mass index; IV: Independent variables; LC-MS/MS: Liquid chromatography tandem mass spectrometry; LLOQ: Lower limit of quantification; P: Phosphorous; PTH: Parathyroid hormone; SGA: Small-for-gestational age; UVB: Ultraviolet B; VDBP: Vitamin D-binding protein; VIF: Variance inflating factor

## Competing interests

The authors declare that they have no competing interests.

## Authors’ contributions

SNK, DGG, AP and DPN designed research; IS, HB, FP and VH conducted research; IS analysed the vitamin D forms; AP analyzed the data; SNK, DGG, AP and DPN wrote the paper; DPN and BCT had primary responsibility for final content. All authors read and approved the final manuscript.

## Supplementary Material

Additional file 1 Descriptive statistics for the primary forms, active forms and epimers of maternal and neonatal 25(OH)D2 and 25(OH)D3. Click here for file

Additional file 2 Correlation matrix (Pearson’s r) for the model predicting neonatal 25(OH)D. Click here for file
