# Supplementary material for: An observational study reveals that neonatal vitamin D is primarily determined by maternal contributions: implications of a new assay on the roles of vitamin D forms
Source: Nutr J. 2013 Jun 7;12:77. doi: 10.1186/1475-2891-12-77 (PMC3680300; doi:10.1186/1475-2891-12-77)
Supplement: Additional file 1 — Descriptive statistics for the primary forms, active forms and epimers of maternal and neonatal 25(OH)D2 and 25(OH)D3. [file 1475-2891-12-77-S1.pdf]

Additional file 1. Descriptive statistics for the primary forms, active forms and epimers of maternal and neonatal 25(OH)D_2 and 25(OH)D_3.

	Vitamin D form	N	Minimum [a]	Maximum	Mean [b]	SD [b]	Mean SEM [c]
Maternal	D_2	60	0.25(3.3%)	73.70	8.64(8.94)	10.24(10.29)	0.18
	D_3	60	0.43(48.3%)	62.25	4.45(8.60)	10.27(13.05)	0.09
	25(OH)D_2	60	1.20(16.7%)	27.12	4.06(4.87)	4.86(4.95)	0.09
	25(OH)D_3	60	1.22(3.3%)	50.50	13.84(14.32)	11.57(11.48)	0.27
	3-epi-25(OH)D_2	60	0.07(26.7%)	23.30	1.99(2.72)	3.55(3.91)	0.09
	3-epi-25(OH)D_3	60	0.00(16.7%)	28.05	2.82(3.38)	4.51(4.74)	0.09
	1α,25$(OH)_2D_2$	60			BLQ (100%)		
	1α,25$(OH)_2D_3$	60	0.04(0.0%)	0.49	0.06	0.06	0.00
Neonatal	D_2	60	1.25(3.3%)	35.90	9.78(10.12)	8.32(8.26)	0.49
	D_3	60	1.81(40.0%)	62.73	5.27(8.79)	10.18(11.95)	0.25
	25(OH)D_2	60	0.23(16.7%)	10.35	2.84(3.41)	2.67(2.56)	0.11
	25(OH)D_3	60	0.31(6.7%)	70.95	13.03(13.97)	12.55(12.48)	0.28
	3-epi-25(OH)D_2	60	0.18(36.7%)	8.75	1.54(2.44)	2.01v(2.05)	0.08
	3-epi-25(OH)D_3	60	0.20(16.7%)	19.28	2.98(3.57)	3.68(3.76)	0.12
	1α,25$(OH)_2D_2$	60			BLQ (100%)		
	1α,25$(OH)_2D_3$	60			BLQ (100%)		

BLQ: below the limit of quantification, SD: standard deviation, SEM: standard error of measurement. All values are expressed in ng/mL.

[a] Based on concentrations above BLQ. Percentages with concentrations BLQ for each analyte are in brackets.

[b] Using a conservative approach, means and SDs are calculated with 0 imputed for BLQ. Means and SDs based purely on concentrations ≥ BLQ are provided in brackets.

[c] Based on two measurements.
