# Supplementary material for: An observational study reveals that neonatal vitamin D is primarily determined by maternal contributions: implications of a new assay on the roles of vitamin D forms
Source: Nutr J. 2013 Jun 7;12:77. doi: 10.1186/1475-2891-12-77 (PMC3680300; doi:10.1186/1475-2891-12-77)
Supplement: Additional file 2 — Correlation matrix (Pearson’s r) for the model predicting neonatal 25(OH)D. [file 1475-2891-12-77-S2.pdf]

Additional file 2. Correlation matrix (Pearson's r) for the model predicting neonatal 25(OH)D.

		Maternal													
		Age	Adjusted BMI	UVB	Ca intake	Vitamin D intake	PTH	Ca	P	$25(OH)D_2$	$25(OH)D_3$	3-epi-25 $(OH)D_2$	3-epi-25 $(OH)D_3$	1α,25 $(OH)_2D_3$	Neonatal 25(OH)D
Maternal	Age	1.000													
	Adjusted BMI	0.122	1.000												
	UVB	-0.304	-0.072	1.000											
	Ca intake	-0.244	0.261	0.378	1.000										
	Vitamin D intake	-0.093	0.096	0.349	0.369	1.000									
	PTH	-0.142	0.315	0.039	-0.007	-0.011	1.000								
	Ca	0.148	-0.004	-0.136	-0.145	-0.086	0.081	1.000							
	P	0.080	0.124	-0.076	-0.092	0.024	0.013	0.571	1.000						
	$25(OH)D_2$	0.047	0.032	0.040	0.112	0.421	0.056	0.097	0.243	1.000					
	$25(OH)D_3$	0.325	0.046	-0.067	0.055	0.037	-0.047	0.107	0.140	0.158	1.000				
	3-epi-$25(OH)D_2$	0.232	0.009	-0.301	-0.019	-0.136	-0.025	0.068	0.134	0.234	0.434	1.000			
	3-epi-$25(OH)D_3$	0.256	0.070	-0.275	0.014	-0.108	-0.063	-0.099	0.109	0.265	0.510	0.858	1.000		
	1α,$25(OH)_2D_3$	0.018	-0.023	-0.137	-0.066	-0.073	-0.040	0.006	-0.048	-0.087	-0.011	-0.077	0.020	1.000	
Neonatal 25(OH)D		-0.038	-0.003	-0.156	0.108	0.180	0.053	-0.005	0.038	0.439	0.437	0.505	0.584	0.000	1.000

BMI: body mass index, Ca: calcium, P: phosphorus, PTH: parathyroid hormone, UVB: ultraviolet B.
